# Small intestinal submucosa promotes angiogenesis via the Hippo pathway to improve vaginal repair

**DOI:** 10.17305/bb.2023.9052

**Published:** 2023-10-01

**Authors:** Yanlai Xiao, Yanpeng Tian, Jingkun Zhang, Qian Li, Wenxin Shi, Xianghua Huang

**Affiliations:** 1 Department of Obstetrics and Gynecology, The Second Hospital of Hebei Medical University, Shijiazhuang, Hebei, China; 2 Department of Obstetrics and Gynecology, The Third Hospital of Hebei Medical University, Shijiazhuang, Hebei, China

**Keywords:** Vaginal reconstruction, small intestinal submucosa (SIS), Hippo pathway, large animals

## Abstract

Vaginal reconstruction has incorporated the use of gastrointestinal segments for decades, but the technique is inevitably associated with complications. Tissue-engineering techniques, however, have brought great hope for vaginal reconstruction. This study aimed to evaluate the utility of small intestinal submucosa (SIS) in reconstructing clinically significant large vaginal defects in a porcine model and to investigate the role of the Hippo pathway in the vascular remodeling process. The composition and mechanical properties of SIS were characterized. Full-thickness vaginal defects were established in 10 minipig donors, with 4-cm lengths removed and replaced by an equal-sized SIS scaffolds. The neovaginas were subjected to macroscopic, histological, immunohistochemical, and molecular evaluations at 4 and 12 weeks after the surgery. Four weeks after the operation, extracellular matrix reorganization and complete coverage of the surface of the luminal matrix by vaginal epithelium were observed, accompanied by the formation of a microvascular network and the regeneration of smooth muscles, albeit disorderly arranged. Twelve weeks after implantation, enhancements were seen in the formation of the multi-layered squamous epithelium, angiogenesis, and large muscle bundle formation in the vagina. Additionally, the expression levels of angiogenesis-related proteins, proliferation-related proteins, and Hippo pathway-related proteins in the neovagina were significantly increased. These results indicate that SIS could be used to reconstruct large vaginal defects and that the vascular remodeling process is potentially regulated by the Hippo pathway.

## Introduction

Mayer–Rokitansky–Kuster–Hauser (MRKH) syndrome affects approximately 1 in 4500–5000 newborn females and is the second leading cause of primary amenorrhea [1]. Currently, the clinical treatment of MRKH can be categorized into non-surgical and surgical treatments. Non-surgical treatment is the first line of treatment recommended by the American College of Obstetrics and Gynecology (ACOG) [2] and satisfactory vaginal length can be obtained without surgery. However, its clinical applicability is limited due to issues, such as lengthy treatment period, intense localized pain, bleeding, and poor patient compliance. Thus, patients often prefer surgical options for vaginal reconstruction. Bowel vaginoplasty is one of the most popular vaginal reconstruction methods, which results in an ideal vaginal length and sexual satisfaction. However, this treatment modality is associated with various complications, such as mucus production, chronic bacteriuria, metabolic disturbance, and potential malignant transformation [3, 4]. Additionally, patients may experience a prolonged hospital stay or impaired functions due to damaged tissues.

In recent years, tissue engineering technology has provided a new direction for organ reconstruction and tissue repair. Scaffolding materials are the most important component of tissue engineering because these biomaterials provide physical support and can also influence cell fate through their inherent physical and chemical properties. Decellularized matrices derived from native tissues, such as small intestinal submucosa (SIS), appear to be an ideal candidate for tissue engineering scaffolds. SIS is composed of more than 90% collagen, with a biomimetic three-dimensional microenvironment, biodegradability, low immunogenicity and is rich in a variety of growth factors, such as platelet-derived growth factor-BB (PDGF-BB), vascular endothelial growth factor (VEGF), epidermal growth factor (EGF), and basic fibroblast growth factor (bFGF) [5, 6], which can promote cell adhesion, proliferation, migration, and differentiation to enhance cell expansion. SIS is an acellular material that has gained much attention for the reconstruction of tissue defects, including skin wound repair [7], hernia repair [8], bladder augmentation [9], chronic vocal fold scar [10], and pelvic organ prolapse [11]. However, the use of the SIS matrix for vaginal reconstruction in large animal models has not been studied.

Insufficient angiogenesis is often associated with poor clinical outcomes when a graft is implanted in vivo. The inability to establish effective microcirculation is an important factor restricting tissue engineering in the treatment of soft tissue injury, especially in large tissue defects [12]. The cell/material interface is a complex and dynamic microenvironment. Biomaterials can determine the fate of cells through their inherent properties (such as hardness, surface structure, pore structure, etc.) [13]. The physical and mechanical signals in the extracellular microenvironment can regulate cell morphology through YAP/TAZ, thereby affecting cell growth, differentiation, and even determining the fate of cells [14–16]. More and more evidence shows that YAP/TAZ plays an important role in angiogenesis. YAP/TAZ regulates the proliferation, migration, and survival of endothelial cells, and then regulates vascular germination, vascular barrier formation, and vascular remodeling [17]. In addition, YAP/TAZ can also inhibit vascular bleeding during development [18]. Therefore, the Hippo signaling pathway plays a key role in this process [19].

This study aimed to explore the application value of SIS in treating large animal models with vaginal defects and the role of the Hippo pathway in vascular reconstruction with SIS, which could be fundamental for clinical application.

## Materials and methods

### Preparation of small intestinal submucosa (SIS)

The preparation of SIS followed a previously described procedure [20]. Briefly, the small intestine was cut into about 20 cm long and washed with a saline solution. First, the serosa and muscular layer were mechanically removed to obtain the submucous membrane. Second, the submucosa was immersed in a solution containing methanol and chloroform (1:1, V/V) in the ventilation cabinet for 12 h. Third, the membrane was incubated in 0.05% trypsin/0.05% ethylenediamine tetraacetic acid at 37 ^∘^C for 12 h. Then, the membrane was treated with 0.5% sodium dodecyl sulfate in 0.9% sodium chloride for 4 h. Finally, the membrane was soaked in 0.1% peracetic acid and 20% ethanol for 30 min. The membrane was freeze-dried and compressed by six layers, vacuum-sealed into hermetic packaging, and terminally sterilized by using gamma irradiation (25KGY).

### Histological analysis

SIS samples of 5 × 5 mm were fixed in 4% paraformaldehyde solution for 48 h at room temperature and subsequently removed to a dehydration box. The dehydration box was successively dehydrated in gradient alcohol. The scaffold was embedded in paraffin, cut into 4-µm thick sections, and stained with hematoxylin-eosin (HE), DAPI staining, Masson’s trichrome staining, and Sirius red staining, according to the manufacturer’s instructions (Servicebio, China), and were then observed under a microscope.

### Scanning electron microscopy

SIS samples of 5 × 5 mm fixed in 2.5% glutaraldehyde at 4 ^∘^C overnight and washed thrice with PBS. The scaffold sections were sequentially immersed in ethanol of different concentration gradients. Then, they were dried using critical point drying, coated with Au, and the surface ultrastructures of both sides were obtained using a scanning electron microscope (SEM; Jeol Corp., Japan) at an accelerating voltage of 10 kV.

### Mechanical properties

SIS specimens were cut into approximately 40 mm × 5 mm strips (*n* ═ 3) in the longitudinal direction. Subsequently, the specimens were incubated in a phosphate buffer solution (pH 7.2–7.4) for 1 h at 37 ^∘^C. Tensile testing (Instron model 3366) was performed by elongating the SIS specimens at a speed of 5 mm/min, and a load cell of 50 N. Stress–strain curves were generated, and the modulus of elasticity for each specimen was determined from the slope of the curve.

### Minipig model of partial vaginectomy

This study used 10 females, sexually mature, 5–6 months old, non-pregnant and without childbirth minipigs, with a body weight of 25–30 kg. The animals were randomly allocated into two groups: one group was implanted with the SIS matrix for 4 weeks (*n* ═ 5), while the other group was implanted for 12 weeks (*n* ═ 5). The grafts were implanted via partial vaginectomy (4 cm), and normal vaginal tissues were excised and used as a control. Before the surgery, SIS was trimmed and soaked in normal saline, sutured in a cylindrical shape, and lined with a mold of an appropriate size to avoid adhesion and contracture. The animals were kept in the horizontal position after successful anesthesia. During the surgery, an incision (10 cm) was made in the middle line of the abdomen to expose the entire length of the vagina. The length of the excised vagina was approximately 4 cm. The upper and lower ends of the graft were anastomosed with the outer orifice of the cervix and the vaginal stump, respectively. The abdomen was closed layer by layer after no active bleeding was observed. After the operation, 3.2 million units of penicillin were injected into the gluteus maximus muscle. All surgeries were performed by the same surgical team and were largely analogous to the real-world clinical condition. The minipigs were housed and fed in separate cages.

### Histological evaluation

The minipigs were sacrificed 4 and 12 weeks after the operation, respectively, and the full-thickness vaginal graft/tissue explants were harvested and embedded in paraffin, then sectioned into 4 µm slices. H&E staining and Masson’s trichrome staining were performed according to the manufacturer’s instructions (Servicebio, China). Immunohistochemical staining was also performed with various primary antibodies, including CK14 (1:400; Servicebio, China), α-actin (1:200; Servicebio, China), CD31 (1:800; Servicebio, China), and PCNA (1:400; Servicebio, China) diluted in antibody diluent at 4 ^∘^C overnight. After that, biotinylated secondary antibody (ZSGB-Bio, China) was applied for 1 h at room temperature and the samples were stained with 3,3-diaminobenzidine (DAB). The coloration was stopped with distilled water, and the nuclei were counterstained with hematoxylin (Servicebio, China). These slides were then examined using a light microscope (ZEISS, Germany).

### Western blotting analysis

Protein was extracted from the neovagina using RIPA lysis buffer (Servicebio, China), and protein concentrations were determined using a BCA Protein Assay Kit (Solarbio, China). The protein samples were denatured at 100 ^∘^C for 10 min. The 10% SDS-PAGE (Biotides, China) was utilized to separate the proteins, which were then transferred to PVDF membranes (Sigma, USA). After blocking with 5% skim milk for 1 h, the PVDF membranes were incubated overnight at 4 ^∘^C with primary antibodies, rabbit polyclonal anti-CK14 (1:1000; Servicebio, China), rabbit polyclonal anti-α-actin (1:1000; Servicebio, China), rabbit polyclonal anti-CD31 (1:2000; Servicebio, China), rabbit polyclonal anti-PCNA (1:2000; Servicebio, China), rabbit polyclonal anti-YAP1 (1:1000; Servicebio, China), rabbit polyclonal anti-TAZ (1:1000; Beyotime, China), rabbit polyclonal anti-TEAD1 (1:500; Servicebio, China), rabbit polyclonal anti-c-Myc (1:500; Servicebio, China), rabbit polyclonal and anti-Cyclin D1 (1:500; Servicebio, China), mouse monoclonal anti-β-tubulin antibody (1:10000; Affbiotech, USA), and rabbit polyclonal anti-GAPDH (1:2000; Servicebio, China). The next day, the blots were washed 3 times for 10 min each using TBST. The membranes were then incubated with goat anti-rabbit IgG (1:5000; Servicebio, China) and rabbit anti-mouse IgG (1:10000; Servicebio, China) for 1 h. Lastly, the blots were imaged with the ChemiDoc MP imaging system (Bio-Rad, USA) to determine the protein expression level.

### Ethical statement

All procedures that involved animals were approved by the Ethics Committee of the Second Hospital of Hebei Medical University (NO. 2020-P018).

### Statistical analysis

Each test was performed at least three times. All results were analyzed by using SPSS 21.0 software (Windows 7). Analysis of variance (ANOVA) tests were performed to evaluate the relationships between the groups. When ANOVA indicated a significant difference, the least significant difference test was used for further comparison between the groups. All data are presented as mean ± SEM. Results are expressed as *P* values with *P* < 0.05 representing statistical significance.

## Results

### Characteristics of small intestinal submucosa (SIS)

**Figure 1.**
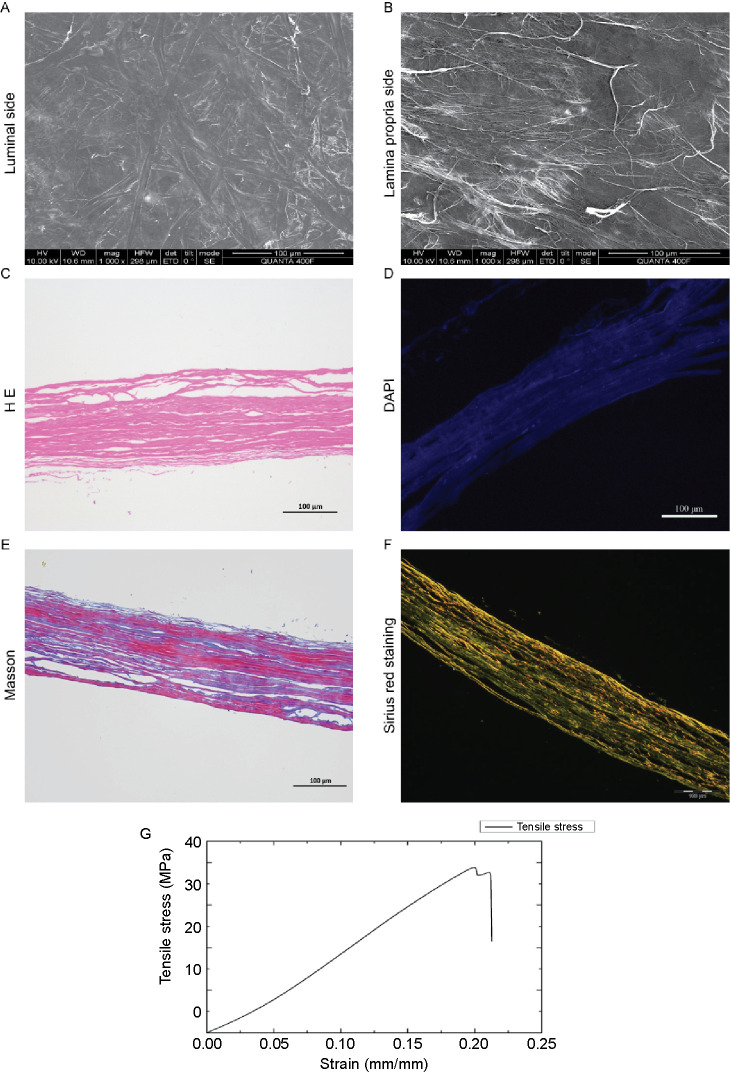
**Scanning electron micrographs, histological analysis, and mechanical properties of SIS.** (A) The luminal side of SIS; (B) The lamina propria side of SIS; (C) HE staining of SIS; (D) DAPI staining of SIS, no nuclear expression; (E) Masson’s trichrome staining of SIS, red colored fibrils depict muscle fibers and blue colored fibrils depict collagen fibers; (F) Sirius red staining of SIS, orange/red colored fibrils depict collagen type I and green colored fibrils represent collagen type III. Scale bar: 100 µm; (G) Stress–strain curves of strip specimens with longitudinal tensile (*n* ═ 3). SIS: Small intestinal submucosa; HE: Hematoxylin-eosin.

The SIS matrix exhibited a reticular appearance on macrography and appeared grayish-white in color. SEM showed that there was no cell debris on either surface of the SIS matrix. The luminal side of the SIS was glossy, and the lamina propria of SIS contained collagen fibers of different diameters, intertwined to form a reticular structure (Figure 1A and 1B). No nuclear material remained in the SIS matrix, as shown by H&E and DAPI staining (Figure 1C and 1D). Masson’s trichrome staining showed the collagen fibers intertwined into a network (Figure 1E). Further analysis using Sirius red staining to further assess these subtypes revealed that SIS was mainly composed of type I collagen fibers, but also contained smaller amounts of collagen type III (Figure 1F). Collagen fibers were identified as the main mechanical component in the acellular matrix [21]. Collagen type I contributed to tensile strength, while collagen type III and elastin provided resilience and elasticity [22, 23].

### Mechanical properties of small intestinal submucosa (SIS)

To further characterize the mechanical properties of SIS, we performed tensile strength assessments using a standard uniaxial tensile test. The stress–strain curve of SIS showed that the initial elasticity and stress increased rapidly. The ultimate tensile strength of SIS was 35.36 ± 2.60 MPa (Figure 1G). Altogether, the results showed that SIS formed a three-dimensional material composed of collagen fibers and showed high deformation resistance behavior.

### Macroscopic and microscopic evaluation

**Figure 2.**
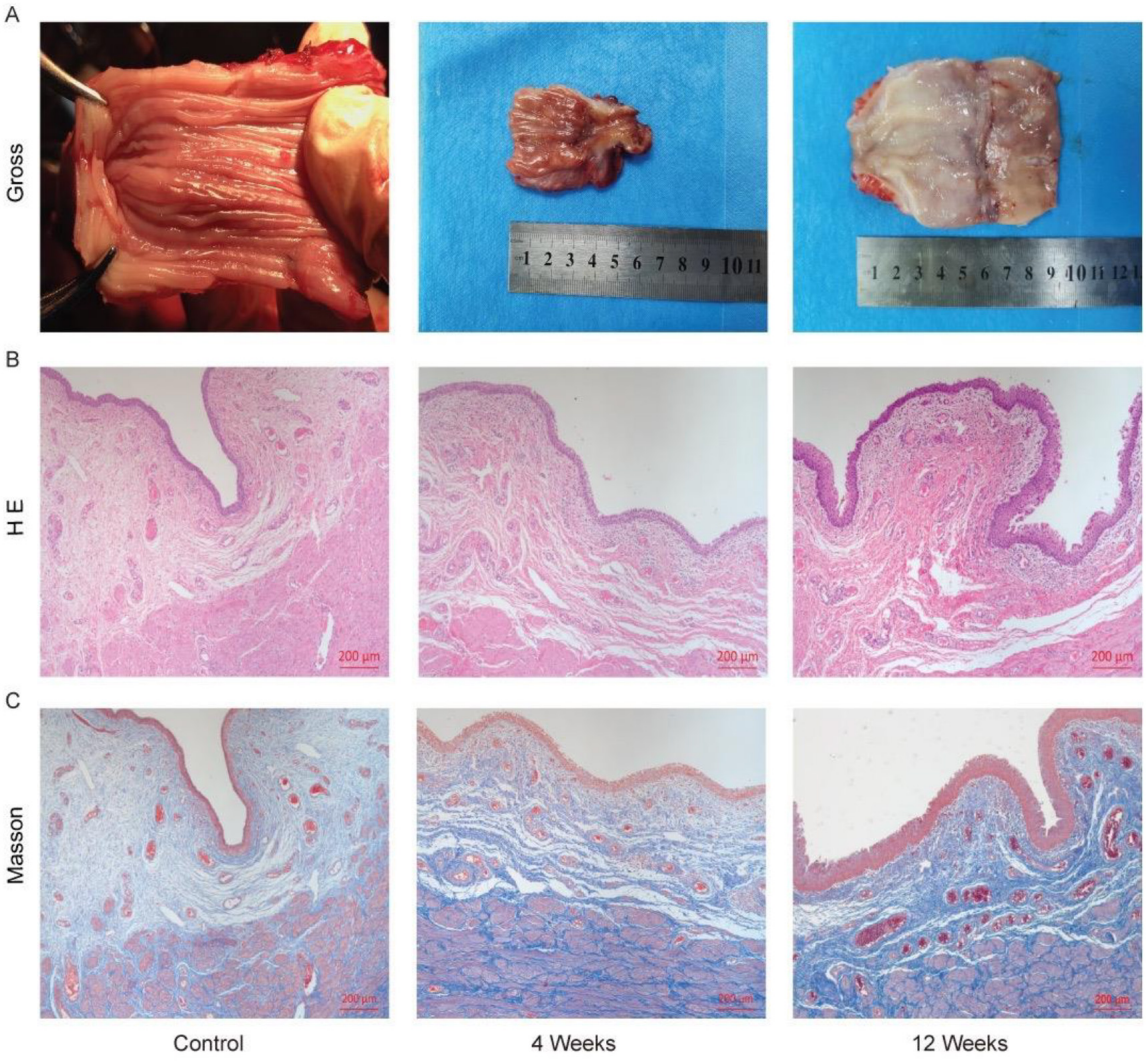
**Gross morphology and histological evaluation of neovagina.** (A) Gross morphology of normal vagina and neovagina at different time points; (B) HE staining of normal vagina and neovagina at different time points; (C) Masson’s trichrome staining of normal vagina and neovagina at different time points, red areas depict epithelium or smooth muscle fibers, blue areas represent collagen fibers. The bar represents 200 µm. HE: Hematoxylin-eosin.

All minipigs survived with a good appetite until their scheduled termination. General observation showed that in the 4-week group, the entire vagina was covered with pink mucosa, the mucous membrane was smooth, and the vaginal muscle layer was thin and inelastic; while in the 12-week group, the vaginal muscle layer was thickened and elastic, similar to the normal vagina (Figure 2A). Microscopically, H&E showed that in the 4-week group, the neovagina had a complete epithelial formation with the epithelial cells arranged in approximately 4–5 layers. The lamina propria was observed under the epidermis, which was loose connective tissue with abundant neovascularization. In the 12-week group, the epithelium was further thickened (Figure 2B). Masson’s trichrome staining showed that the smooth muscle layer was formed in both groups, and the muscle bundles were arranged irregularly in the 4-week group. However, in the 12-week group, the muscle bundles were arranged relatively regularly, similar to normal vaginal tissue (Figure 2C).

### Epithelial, smooth muscle bundles regeneration and vascularization in neovagina

**Figure 3.**
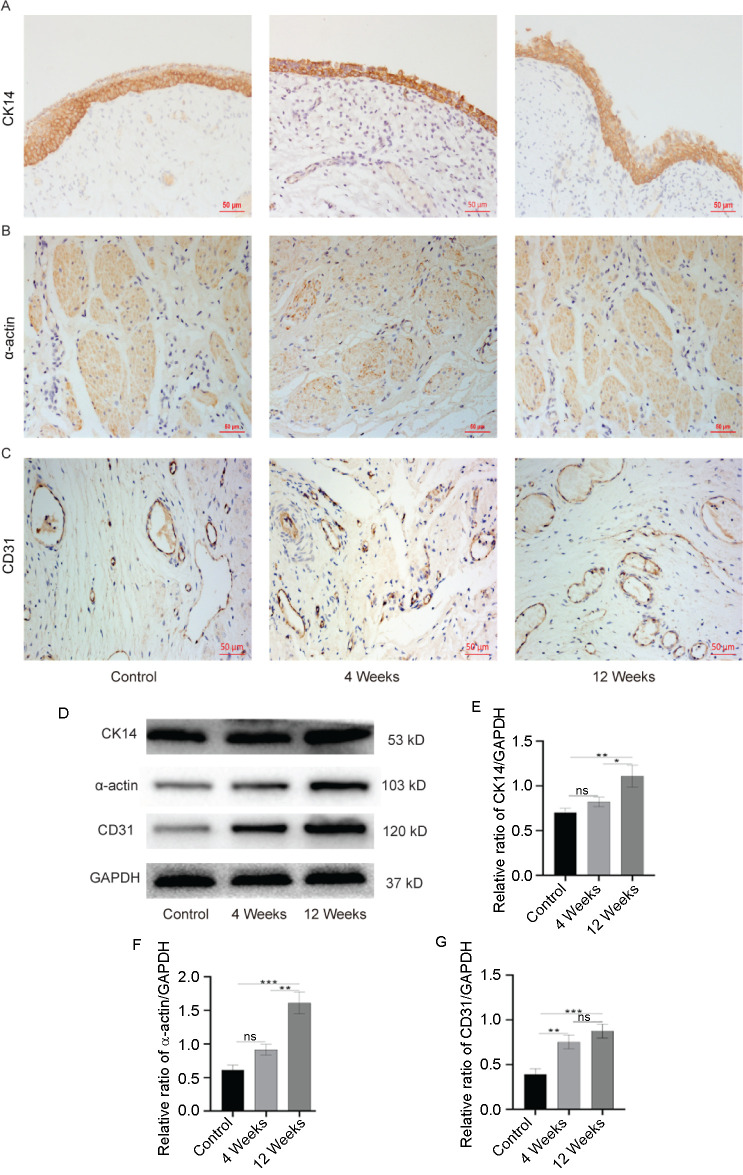
**Immunohistochemistry and specific protein expression of neovagina.** Immunohistochemistry for (A) CK14, (B) α-actin, and (C) CD31. Scale bar: 50 µm. Specific protein expression of neovagina; (D) Representative immunoblots of CK14, α-actin, and CD31, using GAPDH as the reference. Quantification of protein expression revealed that the expression levels of (E) CK14 and (F) α-actin in 4-week group were similar to that in the control group, but significantly increased in 12-week group. The activity of (G) CD31 in 4-week and 12-week group were higher than that in the control group. Data are presented as the mean ± SEM. *P < 0.05, **P < 0.01, ***P < 0.001, ns, no significance. Data are representative of three independent experiments in each group. CK14: Cytokeratin 14.

Immunohistochemical staining of cytokeratin 14 (CK14) showed that 4–5 layers of squamous epithelium formed in the neovagina at 4 weeks after the operation, with gradual thickening over time, and was similar to that of the normal vagina at 12 weeks (Figure 3A). Smooth muscle cells were detected in the neovagina through immunohistochemical staining of α-actin. Irregularly arranged smooth muscle bundles were found in the 4-week group, and they became more regular in the 12-week group (Figure 3B). Immunohistochemical staining of CD31 showed that a large number of new immature small blood vessels were formed in the 4-week group, and more mature blood vessels were formed at 12 weeks after surgery (Figure 3C). Western blot analyses were performed to quantify these parameters (Figure 3D). The expression levels of CK14 and α-actin in the 4-week group were increased, but there was no significant difference compared with the control group (*P* > 0.05) (Figure 3E and 3F). The expression levels of CK14 and α-actin in the 12-week group were significantly higher than in the control group (*P* < 0.05). Interestingly, the expression level of CD31 in the 4-week group was significantly higher than in the control group (*P* < 0.05), and further increased in the 12-week group (*P* < 0.01) (Figure 3G).

### Promoting endothelial cells proliferation of neovagina in vivo via the Hippo pathway

**Figure 4.**
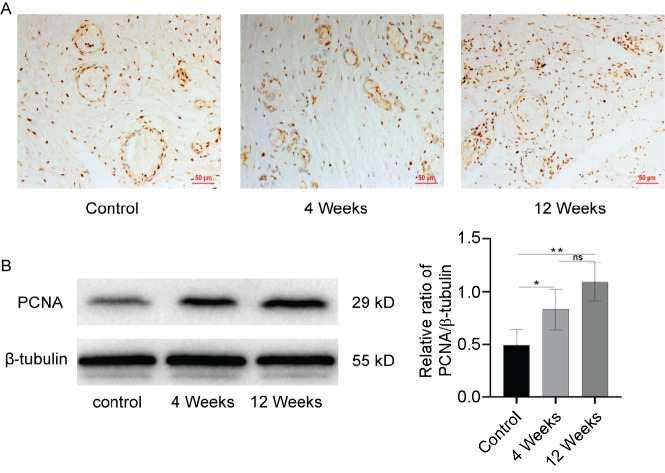
**Immunohistochemistry and protein expression of PCNA in neovagina.** (A) Immunohistochemistry for PCNA. Scale bar: 50 µm; (B) Representative immunoblots of PCNA, using β-tubulin as the reference. Quantification of protein expression revealed that the expression levels of PCNA in 4-week and 12-week group were higher than in the control group. Data are presented as the mean ± SEM. *P < 0.05, **P < 0.01, ***P < 0.001, ns, no significance. Data are representative of three independent experiments in each group. PCNA: Proliferating cell nuclear antigen.

**Figure 5.**
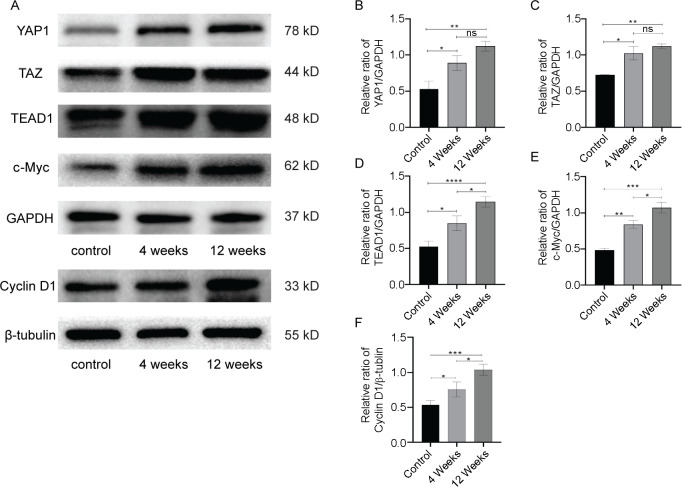
**Specific protein expression of the Hippo pathway.** (A) Representative immunoblots of YAP1, TAZ, TEAD1, and c-Myc, using GAPDH as the reference. Representative immunoblots of cyclin D1, using β-tubulin as the reference. Quantification of protein expression revealed that the expression levels of (B–F) YAP1, TAZ, TEAD1, c-Myc, and cyclin D1 in 4-week and 12-week group were higher than that in the control group. Data are presented as the mean ± SEM. *P< 0.05, **P < 0.01, ***P< 0.001, ****P < 0.0001, ns, no significance. Data are representative of three independent experiments in each group.

Proliferating cell nuclear antigen (PCNA) is a predictable molecular marker for detecting proliferative cells. Immunohistochemical staining of PCNA showed that a large number of proliferating cells were expressed in and around the blood vessels of the neovagina in both groups (Figure 4A). Western blot analyses showed that the expression level of PCNA in the 4-week group was significantly higher than in the control group (*P* < 0.05) and further increased in the 12-week group (*P* < 0.01) (Figure 4B). To determine the role of the Hippo signaling in angiogenesis, a western blot was used to detect key molecules of the Hippo pathway and cell cycle proteins. The expression levels of YAP1, TAZ, TEAD1, c-Myc, and cyclin D1 were significantly increased in the 4-week group compared with the control group (*P* < 0.05) and were further elevated in the 12-week group (*P* < 0.01) (Figure 5A–5F). The expression trend of these proteins is consistent with that of CD31. These preliminary findings indicated that the Hippo pathway potentially regulates endothelial cell proliferation in the neovagina.

## Discussion

SIS has been successfully used in many cases due to its low immunogenicity and excellent biocompatibility. It is generally accepted that a complete removal of xenogenic cell material from the scaffold is the main requirement for the tolerability of xenogenic scaffolds [24]. In this study, SEM and H&E staining showed no residual cell material in the SIS matrix, indicating that SIS provided a natural environment for host cells to migrate and promoted tissue generation without immune rejection. SEM showed that the luminal side of the SIS was glossy, and the lamina propria of SIS had collagen fibers of different diameters that intertwined to form a reticular structure, which is extremely advantageous for cell adhesion, growth, and proliferation [25]. The poor mechanical properties of SIS scaffolds may limit their applications, but they could be adjusted by applying multiple layers [18]. The ultimate tensile strength of this graft was much higher than that of our prefabricated SIS and commercially available mesh (Surgisis) [5, 26], as it consists of six laminates. In order to improve the mechanical properties and stabilized functionalities of SIS, Zhang et al. [27] used the Procyanidins crosslinked small intestine submucosa (PC-SIS) to repair the full-thickness bladder defects in a rabbit model. They demonstrated that PC-SIS scaffolds can rapidly promote in situ tissue regeneration, especially smooth muscle remodeling, which is similar to our results.

SIS has been used to reconstruct a variety of tubular tissues, such as blood vessels [28], ureter [29], and esophageal reconstruction [30]. However, vaginal reconstruction has mainly been performed on small animal models [5, 31], which have significant differences from humans in terms of vaginal tissue length, thickness, and structure. Sheep are often used in pelvic system studies involving vaginal tissue, but spontaneous vaginal prolapse can occur in sheep [32, 33], and prolapse of neovagina is one of the common complications after reconstruction, making them unsuitable for use as large animal models of vaginal loss. Minipigs, on the other hand, are closer to humans in genetics, physiology, metabolism, anatomy, and body size [34, 35]. Therefore, minpigs can be used as large animal models for preclinical research and are widely used in tissue engineering and regenerative medicine [36–38]. In this study, the SIS matrix was implanted in minipigs, eventually promoting vaginal regeneration, including ECM remodeling, angiogenesis, multi-layered squamous epithelium, and large muscle bundles, which were confirmed by H&E, Masson’s trichrome, and immunohistochemical staining. These results suggest that SIS could be used to repair large vaginal deficits.

The degree and speed of mucosification of neovagina are one of the key factors affecting the success of reconstruction, so epithelialization of reconstructed tissues should be realized as soon as possible. The previous experiment of our group showed that after vaginal reconstruction in rats with the printed biomimetic 3D vagina tissues, the epithelial cell layer was approximately 2 to 3 layers after 4 weeks [39]. Other studies have shown that bladder reconstruction with bladder acellular matrix can complete the formation of a single layer of urothelium at 2 weeks after surgery, and a multilayered urothelium formed at 4 weeks after surgery [40]. Our results demonstrated that the epithelial cell layer was approximately 4–5 layers at 4 weeks after surgery, which is important to reduce the occurrence of complications, such as stricture and scarring [41].

The microvascular formation is essential during vaginal regeneration. The early establishment of a rapid and complete network of blood vessels to supply nutrients and oxygen promotes cell survival and vaginal regeneration. The importance of a fast and efficient vascularization for tissue regeneration has been well recognized, and many efforts have been made for scaffold functionalization, such as applying growth factors [42], co-implantation of biological scaffolds with mesenchymal stem cells [39, 43, 44], and revascularization of tissue-engineered grafts [45]. Each strategy has its advantages and disadvantages. However, the clinical application of these techniques still requires more extensive and in-depth research [46, 47]. A previous study reported that SIS contained factors that could promote angiogenesis, such as VEGF and PDGF-BB, and the use of SIS alone for vaginal reconstruction demonstrated angiogenic potential [5]. Our study showed that SIS had a pronounced pro-vascular proliferative effect in vivo, as demonstrated by immunohistochemistry and western blotting of the vascular endothelial cell marker CD31.

The Hippo signaling pathway regulates numerous biological processes, including cell growth, organ size control, and regeneration [48]. In vertebrates, YAP/TAZ is the major downstream effector of the Hippo pathway signaling [49]. YAP/TAZ localization can be cytoplasmic or nuclear. When Hippo signaling is deactivated, YAP/TAZ is dephosphorylated and translocated to the nucleus, where it binds to TEAD and activates the transcription of downstream target genes, such as *CTGF*, *CYR61* and c-Myc [50]. Previous studies showed that YAP/TAZ plays an important role in tissue repair and regeneration, such as hepatocyte regeneration after partial hepatectomy [51], and regeneration after intestinal epithelium, skin, and heart injury [52–54], YAP/TAZ is also a key regulator of vascular sprouting and angiogenesis [55]. Studies have shown that the expression levels of YAP/TAZ are significantly increased during the early development and differentiation of mouse retinal vessels [56]. Kim et al. reported that YAP/TAZ can regulate actin cytoskeleton rearrangement, leading to metabolic changes in endothelial cells during angiogenesis and subsequently affecting the formation of tip endothelial cells. It is also shown that YAP/TAZ can not only promote the proliferation and metabolism of vascular endothelial cells but can also promote the expression of MYC in vascular endothelial cells. It is suggested that YAP/TAZ may regulate the proliferation and metabolism of stalk vascular endothelial cells through the MYC signal pathway during angiogenesis and sprouting [57]. In addition, biomaterials with high mechanical properties could promote the germination of endothelial cells, while YAP/TAZ remained in the cytoplasm after treatment with YAP inhibitor Vitephene, which significantly reduced the number of germinated blood vessels and the invasion distance of new vessels [58]. In this study, proteins representing the establishment of microcirculation (YAP1, TAZ, and TEAD1) and the proliferation of vascular endothelial cells (c-Myc, cyclin D1, and PCNA) were significantly enhanced in both vaginal reconstruction groups. Further, these findings demonstrated that SIS could promote endothelial cell proliferation in reconstituted tissues through the Hippo signaling pathway.

## Conclusion

In this study, we demonstrated successful vaginal reconstruction following SIS implantation in large animals and confirmed that the Hippo signaling pathway might be involved in the angiogenesis of the neovagina. This study provides fundamental preclinical data for the clinical translation of bioengineered grafts However, a major limitation of this study is the relatively small sample size of five animals per group. Additionally, the specific mechanism via which SIS promotes endothelial cell proliferation needs to be further studied in vitro.
